# Caesarean sections and risk of wheezing in childhood and adolescence: data from two birth cohort studies in Brazil

**DOI:** 10.1111/j.1365-2222.2010.03611.x

**Published:** 2011-02

**Authors:** A M B Menezes, P C Hallal, A M Matijasevich, A J D Barros, B L Horta, C L P Araujo, D P Gigante, I S Santos, G Minten, M R Domingues, S C Dumith, F C Barros

**Affiliations:** Postgraduate Programme in Epidemiology, Federal University of PelotasPelotas, Brazil

**Keywords:** adolescent, caesarean section, child, cohort studies, current wheezing, developing countries, epidemiology, persistent wheezing

## Abstract

**Background:**

There is evidence from two meta-analyses that children born through caesarean section (C-section) may have an increased risk of developing asthma compared with those born through vaginal delivery.

**Objective:**

To evaluate the association between mode of delivery and wheezing (current and persistent) in childhood and adolescence, in two birth cohort studies in Brazil.

**Methods:**

The outcome variable was based on the International Study of Allergy and Asthma questionnaire, which collects information about wheezing within the 12 months before the interview. Persistent wheezing was defined when it was present in more than one follow-up at different ages, in the 1993 cohort. The questions were asked to mothers when children were aged 4 years (1993 and 2004 cohorts) and directly to cohort participants at 11 and 15 years (1993 cohort). Mode of delivery was collected by the research team of each cohort when children were born.

**Results:**

Response rates in the last follow-up visit of the 1993 and 2004 cohorts were 85% and 92%, respectively. The prevalence of current wheezing increased from 20% to 28% at 4 years from 1993 to 2004; at 11 and 15 years, the prevalence was around 14% and 12%, in the 1993 cohort. The proportion of C-sections increased from 30.5% to 45% between 1993 and 2004. In each cohort, the prevalence of current wheezing was similar among children born through vaginal and C-section. The risk for persistent wheezing in the 1993 cohort was higher among girls born through C-section than boys.

**Conclusion:**

Despite the increase in the proportion of C-section in two cohorts in Southern Brazil, we found no evidence of an association between mode of delivery and the subsequent risk of wheezing. Among girls, although there was no statistical significance, the risk was higher for those born by C-section, especially regarding persistent wheezing.

## Introduction

Caesarean sections (C-sections) are estimated to be required in 5–15% of all deliveries [1]. Wide differences in the prevalence of these operations are described in low- and middle-income countries: a study showed that in 20 out of 42 countries, C-sections were performed in <1% for the poorest quintile of women, whereas an excess of operative deliveries was shown in seven countries, mostly from Latin America [2]. Lack of C-section when they are needed, are associated with an increased mortality of mothers and babies [1], and excess rates without medical indication are also linked to an increased morbidity and mortality [3].

There is evidence from two meta-analyses [4, 5] that children born through C-section may have an increased risk of developing asthma in comparison with those born through vaginal delivery. Pooled effects were 1.18 and 1.20 in the two available meta-analyses [4, 5]. Most studies included in the meta-analyses, however, were carried out in high-income countries, thus limiting the extrapolation of the findings to low- and middle-income societies.

In Pelotas, a southern Brazilian city, an epidemic of C-sections was reported some years ago [6, 7], and the existence of two population-based birth cohort studies [8, 9] enables us to explore this association using point prevalence of current wheezing at different ages. Not only Brazil has a high percentage of C-sections but also its prevalence of symptoms of asthma is as high as reported from known high-prevalence countries such as Australia, New Zealand and United Kingdom [10].

The aim of the present study was to evaluate the association between mode of delivery and current wheezing or persistent wheezing in childhood and adolescence in two birth cohort studies in Brazil. Our original hypothesis was that individuals born through C-section would be at greater risk of wheezing in comparison with those born through vaginal delivery.

## Material and methods

Pelotas is a 340 000 inhabitant city located in the extreme south of Brazil. Two birth cohort studies were launched in the city within the interval of 11 years [8, 9]. The 1993 Pelotas birth cohort and the 2004 Pelotas birth cohort comprised, respectively, 5249 and 4288 individuals. Both cohorts included all children delivered in Pelotas in these respective years. In each study, mothers were approached at the hospital, and a structured interview was carried out. Subjects belonging to each cohort were re-visited at several occasions. Detailed methodological information on the Pelotas birth cohort studies is available elsewhere [8, 9].

The outcome variable current wheezing was defined based on the International Study of Allergy and Asthma (ISAAC) questionnaire [10–12], which collects information about wheezing through the following question: did you have wheezing within the last 12 months? The questions were asked to mothers when children were aged 4 years (1993 and 2004 cohorts) and directly to cohort participants at 11 and 15 years (1993 cohort). Persistent wheezing was defined by the presence of wheezing in more than one follow-up in the 1993 cohort (at 4 and 11 years and at 11 and 15 years). It was not possible to analyse wheezing at 4, 11 and 15 years due to the small number of cases. Mode of delivery was collected by the research team of each cohort when children were born.

Confounding variables were defined on the basis of the current literature on the association between mode of delivery and current and persistent wheezing [4, 5, 13–21]. These include child, parental and family variables.

*Child variables*: Birth weight (measured by the research team at the time of delivery), sex, gestational age (based on the date of the last period), intrauterine growth restriction (birth weight for gestational age <10th percentile based on the Williams' curves) [22], skin colour (as observed by the interviewer) and birth order (maternal report).

*Maternal variables:* Age and education at the time of delivery, smoking during pregnancy (yes/no, regardless of the amount), smoking at the age of follow-up (except for the 2004 cohort, when smoking was collected when children were aged 12 months) and current wheezing (self-report).

*Paternal variables*: Smoking at the age of follow-up and current wheezing (self-report). These variables were collected for the person living with the mother, regardless of being the natural father of the cohort member or not.

*Family variables*: Socio-economic position was based on the possession of different household assets. Continuous variables were created using principal component analyses. This variable was collected at birth, and divided into tertiles for stratified analyses.

## Statistical analyses

Initial statistical analyses included the calculation of the prevalence and respective 95% confidence intervals (CI) of current wheezing according to type of delivery. Later, the odds ratios (ORs) were calculated in a logistic regression adjusted for confounding variables, using the statistical package Stata version 9.2 (StataCorp, College Station, TX, USA).

In all analyses, the vaginal mode of delivery was set as the reference category. The visit at 4 years of age of the 1993 cohort was weighed given the oversampling of low birth weight children [9]. Analyses were later stratified by sex and socio-economic position at birth [23], and tests for interaction were performed.

The Federal University of Pelotas Medical School Ethics Committee approved all phases of each cohort. Informed consent was obtained from parents.

## Results

Response rates in the last follow-up visit of the 1993 and 2004 cohorts were, respectively, 85% and 92%. In Table 1, we present descriptive data on the outcome variable, exposure of interest and confounding factors. The prevalence of current wheezing increased from 20% to 28% at 4 years from 1993 to 2004. In the 1993 cohort – at 11 and 15 years old – the prevalence was 13.5% and 12%, respectively; persistent wheezing was less prevalent: 6% for those who reported wheezing at 4 and 11 years, 4.5% for those who reported wheezing at 11 and 15 years old and 2.7% for those who reported wheezing at all follow-ups.

**Table 1:** Description of the two birth cohort studies in Pelotas, Brazil

	Birth cohort	
	
Variables	1993 (*n=*5249)	2004 (*n=*4288)
% Current wheezing		
4 years	20.2	27.8
11 years	13.5	NA
15 years	12.1	NA
% Persistent wheezing		
4–11 years	6.2	NA
11–15 years	4.5	NA
4, 11 and 15 years	2.7	NA
Mode of delivery		
Vaginal	69.5	54.8
C-section	30.5	45.2
Sex		
Males	48.8	51.9
Females	51.2	48.1
Birth weight, g (mean ± SD)	3176 (521)	3172 (530)
Gestational age, weeks (mean ± SD)	38.1 (1.6)	38.5 (2.3)
Intrauterine growth restriction		
No	90.4	87.6
Yes	9.6	12.4
Skin colour		
White	64.0	71.1
Black	32.3	10.7
Others	3.7	18.2
Birth order		
First born	35.4	39.3
Second born	27.8	26.6
Third born	17.6	16.5
Fourth born or more	19.2	17.6
Maternal age (mean ± SD)	26.1 (6.4)	26.2 (6.8)
Maternal schooling (mean ± SD)	6.7 (3.5)	8.1 (3.4)
Maternal smoking in pregnancy		
No	66.7	72.8
Yes	33.3	27.2
Maternal smoking after pregnancy		
No	68.2	70.5
Yes	31.8	29.5
Maternal current wheezing		
No	84.3	79.2
Yes	15.7	20.8
Paternal smoking		
No	62.2	73.0
Yes	37.8	27.0
Paternal current wheezing		
No	NA	83.6
Yes	NA	16.4

NA, not available.

The proportion of C-sections increased from 30.5% to 45% between 1993 and 2004. Mean birth weight was stable over time. Mean gestational age was slightly reduced, as well as the proportion of intrauterine growth restriction. Mean maternal age remained almost the same over time, and maternal schooling increased considerably in the 11-year period. Maternal smoking during pregnancy decreased from 33.3% to 27.2%, whereas maternal current wheezing increased from 15.7% to 20.8%, as is shown in Table 1.

Table 2 presents the prevalence of current wheezing in the 1993 and 2004 cohorts and persistent wheezing in the 1993 cohort according to mode of delivery. The prevalence of current and persistent wheezing was remarkably similar among children born through vaginal and C-section at all ages. The proportion of current wheezing at 4 years of age increased by 37% and 41% from 1993 to 2004 among those born through C-section and vaginal delivery, respectively. In the 1993 cohort, the prevalence of current wheezing decreased from childhood to adolescence. In Table 3, adjusted ORs for current and persistent wheezing among children born through C-section are shown. None of them were statistically significant. Maternal asthma was the most important confounding factor in the adjusted analysis, followed by birth weight. The lack of association between mode of delivery and current or persistent wheezing was confirmed in the analyses stratified by sex. No significant interactions were detected (Table 4), although the ORs for persistent wheezing among girls born through C-section were higher than among boys.

**Table 2:** Prevalence (95% confidence interval) of current and persistent wheezing at different ages according to type of delivery: unadjusted analyses (Pelotas, Brazil)

	Type of delivery		
	
	C-section % of wheezing (95% CI)	Vaginal % of wheezing (95% CI)	*P*
2004 cohort			
4 years of age (*n*=3798)	26.8 (24.7; 28.9)	28.7 (26.7; 30.7)	0.20
1993 cohort			
4 years of age (*n*=1270)*	19.5 (15.5; 23.4)	20.4 (17.8; 23.1)	0.70
11 years of age (*n*=4423)	14.4 (12.5; 16.3)	13.1 (11.9; 14.2)	0.24
4 and 11 years (*n*=1168)*	6.4 (3.8; 9.0)	6.2 (4.5; 7.8)	0.88
15 years of age (*n*=4325)	12.1 (10.3; 13.8)	12.1 (11.0; 13.3)	0.97
11 and 15 years (*n*=4225)	5.2 (4.0; 6.4)	4.2 (3.5; 5.0)	0.18

* Analyses weighed for low birth weight.

**Table 3:** Odds ratios (OR) for wheezing at different ages according to type of delivery (vaginal is the reference group): adjusted analyses (Pelotas, Brazil)*

	OR (95% CI)	*P*
2004 cohort		
4 years of age (*n*=2974)	0.96 (0.81; 1.15)	0.66
1993 cohort		
4 years of age (*n*=1215)†	1.16 (0.81; 1.68)	0.41
11 years of age (*n*=3210)	1.18 (0.94; 1.48)	0.16
4 and 11 years (*n*=855)†	1.23 (0.62; 2.47)	0.55
15 years of age (*n*=3086)	1.02 (0.80; 1.31)	0.85
11 and 15 years (*n*=3083)	1.19 (0.81; 1.75)	0.37

* Adjusted for child (sex, birth weight, gestational age, intra-uterine growth restriction, skin colour and birth order), maternal (age, education, smoking during pregnancy and afterwards, and current wheezing), paternal (smoking and current wheezing) and family (socio-economic position) variables.

† Analyses weighed for low birth weight.

CI, confidence interval.

**Table 4:** Odds ratios (OR) for wheezing at different ages according to type of delivery (vaginal is the reference group): adjusted analyses stratified by sex (Pelotas, Brazil)*

	Boys		Girls	
	
	OR (95% CI)	*P*	OR (95% CI)	*P*
2004 cohort				
4 years of age	1.04 (0.81; 1.32)	0.77	0.88 (0.68; 1.14)	0.34
1993 cohort				
4 years of age†	1.18 (0.71; 1.96)	0.54	1.12 (0.66; 1.91)	0.67
11 years of age	1.15 (0.84; 1.58)	0.38	1.19 (0.85; 1.67)	0.30
4 and 11 years†	0.95 (0.43; 2.07)	0.89	1.54 (0.51; 4.64)	0.44
15 years of age	0.78 (0.53; 1.14)	0.20	1.25 (0.90; 1.74)	0.19
11 and 15 years	0.92 (0.53; 1.60)	0.78	1.59 (0.93; 2.71)	0.09

* Adjusted for child (birth weight, gestational age, intra-uterine growth restriction, skin colour and birth order), maternal (age, education, smoking during pregnancy and afterwards, and current wheezing), paternal (smoking and current wheezing) and family (socio-economic position) variables.

† Analyses weighed for low birth weight.

CI, confidence interval.

The same was observed for socio-economic position (Table 5), i.e. none of them were statistically significant. However, the risk for persistent wheezing (at 11 and 15 years) among the wealthiest born through C-section was 82% higher than among less wealthy subjects, with a borderline *P*-value (0.06).

**Table 5:** Odds ratios (OR) for wheezing at different ages according to type of delivery: adjusted analyses stratified by tertiles of socio-economic position (Pelotas, Brazil)*

	First tertile (poorest)		Second tertile (intermediate)		Third tertile (wealthiest)	
	
	C-section, OR (95% CI)	*P*	C-section, OR (95% CI)	*P*	C-section, OR (95% CI)	*P*
2004 cohort						
4 years of age	0.74 (0.55; 0.99)	0.05	1.23 (0.91; 1.65)	0.17	0.83 (0.60; 1.16)	0.28
1993 cohort						
4 years of age†	1.09 (0.65; 1.84)	0.75	1.06 (0.51; 2.20)	0.87	1.16 (0.54; 2.49)	0.71
11 years of age	1.28 (0.89; 1.85)	0.18	1.02 (0.65; 1.55)	0.94	1.32 (0.86; 2.01)	0.20
4 and 11 years†	1.19 (0.38; 3.75)	0.77	2.17 (0.36; 12.9)	0.39	0.68 (0.16; 2.84)	0.60
15 years of age	0.94 (0.62; 1.42)	0.76	0.97 (0.61; 1.55)	0.89	1.14 (0.72; 1.79)	0.58
11 and 15 years	1.12 (0.55; 2.24)	0.76	0.80 (0.37; 1.73)	0.57	1.82 (0.98; 3.36)	0.06

* Adjusted for child (birth weight, gestational age, intra-uterine growth restriction, skin colour and birth order), maternal (age, education, smoking during pregnancy and afterwards, and current wheezing), and paternal (smoking and current wheezing) variables.

† Analyses weighed for low birth weight.

## Discussion

Using data from two population-based cohorts in southern Brazil, we found no evidence of a significant association between mode of delivery and the subsequent risk of wheezing. This relationship has been debated over the last years. Most original studies available found non-significant results, or slightly higher risk of asthma among those born through C-sections compared with those born by vaginal delivery [13–21]. Data from low- and middle-income countries on this association are virtually non-existent. Most recently, two meta-analyses were carried out on this topic, and both concluded that there is a moderate increase (18–20%) in the risk of asthma among subjects born through C-section [4, 5]. As only 1.5% of cases of asthma were attributable to delivery by C-section in the meta-analyses from Bager et al. [4], the authors believe it is unlikely that the increased use of C-section during the last decades contributed much to the increase of asthma in the same period.

There is a biological plausibility for an association between mode of delivery and asthma, because the development of the immune system may differ according to the type of delivery. Special attention has been paid to the production of types 1 and 2 T-helper cytokines [18]. However, in addition to biological factors, the environment may play a role, and therefore, studies in low- and middle-income settings, such as ours, are desirable, mainly because, differently from what is reported in some high-income countries, asthma in Brazil is more frequent among subjects from low socio-economic groups [24, 25]. In Pelotas, the proportion of deliveries through C-section increased from 30.5% to 45% from 1993 to 2004 [8]. This proportion reaches 82%, if we consider only private deliveries (paid for out of pocket by patients). Because in Brazil only rich families pay for their deliveries out of pocket, exposure to C-section is much more frequent among the rich [8]. In order to overcome the possible confounding by socio-economic status in our analyses, we first adjusted the association between mode of delivery and wheezing to socio-economic position and maternal schooling. Because residual confounding could still be present using this approach, we also stratified our analyses by socio-economic position. Both approaches failed to modify the results, suggesting that the explanation for our null findings is not being confounded by socio-economic position. Despite the marked increase of 48% in C-sections between the 1993 and 2004 cohort in Southern Brazil, we did not find a statistical association between C-section and the presence of wheezing in the last 12 months.

Some methodological issues need to be highlighted. First, we rely on prospective data of two well-known population-based birth cohort studies [8, 9]. Second, by assessing the outcome at different ages, we minimize the likelihood of spurious findings. Third, data on the exposure were actively collected by the research team, data on the outcome were collected using an internationally accepted and validated questionnaire (ISAAC), and information on confounders was also prospectively obtained using standardized methods.

We also would like to point out some limitations of our study. We do not know whether C-sections in these two Brazilian cohorts were carried out by ‘doctor indication’ or due to other factors, such as doctor or patients convenience; it appears to us that the great majority of C-sections in our place are not a well-defined medical indication and most of these C-sections occur among rich families. This is relevant because in one of the meta-analyses the association between C-section and asthma decreased and was no longer significant when exposure was defined as birth by an ‘uncontaminated’ C-section (OR=1.17%, 95% CI 0.81–1.69) [19]. We were not able to perform our analysis taking into account the type of C-section due to the absence of this information.

It could be argued that statistical power was also a limitation of our study. It was not possible to analyse persistent wheezing at the age of 4, 11 and 15 years old for the 1993 cohort stratified by sex and particularly by socio-economic position; the sample size was not enough for this analyses. We decided to maintain the stratification by sex due to the fact that the association found by Renz-Polster was gender specific, with a positive association only among girls (OR=1.53%, 95% CI 1.11–2.10) and not among boys (OR=1.08%, 95% CI 0.81–1.43) [19]. We also found a higher risk for wheezing among girls born by C-section than boys, and especially persistent wheezing, but this did not reach significance. We cannot be sure whether or not this was due to sample size and its statistical power.

In conclusion, some findings of the present study were very similar to those presented in two recent meta-analyses [4, 13]. For example, in the 1993 cohort at the age of 4 and 11 years, we found an increased risk of current wheezing among those born through C-section of 16% and 18%, respectively, close to the 18–20% reported in the two meta-analyses. However, our effect measures (OR) were not significant, as well as other studies included in the meta-analyses, which also found similar effects, but without statistical significance. This might reinforce the hypothesis of insufficient statistical power to detect these associations.

A point to be highlighted in our study is that, regardless of the similar assessment of current wheezing across all ages, there was a lack of pattern in our results. For example, sometimes the OR of current wheezing among those born through C-section was higher than the unity, indicating an increased risk, but in other cases it was lower than the unity, indicating a decreased risk. This might suggest that the association between current wheezing and mode of delivery is not evident in Brazil. On the other hand, it appears that severe wheezing such as persistent wheezing, and particular among girls may be associated with C-sections. Other studies in developing countries are needed to confirm our findings.
